# Association between heart rate-corrected QT interval and severe peripheral arterial disease in patients with type 2 diabetes and foot ulcers

**DOI:** 10.1530/EC-21-0140

**Published:** 2021-07-05

**Authors:** Jing Hong, Wen-Yue Liu, Xiang Hu, Fei-Fei Jiang, Ze-Ru Xu, Fang Li, Fei-Xia Shen, Hong Zhu

**Affiliations:** 1 Department of Endocrinology, The First Affiliated Hospital of Wenzhou Medical University, Wenzhou, China; 2 Department of Endocrinology, Ruian Traditional Chinese Medicine Hospital, Wenzhou, China

**Keywords:** diabetes, foot ulcer, QTc interval, peripheral arterial disease

## Abstract

**Background:**

A prolonged heart rate-corrected QT interval (QTc) has been associated with peripheral artery disease (PAD) in the general population. However, no study to date has identified a link between prolonged QTc and the severity of PAD in patients with diabetes mellitus and foot ulcers (DFUs). This study aimed to investigate this relationship.

**Methods:**

This multicenter study enrolled 281 patients with DFUs. The severity of PAD was classified into no severe PAD group (without stenosis or occlusion) and severe PAD group (with stenosis or occlusion) based on duplex ultrasonography. The association of prolonged QTc with severe PAD was evaluated in a multivariable mixed-effect logistic regression model, with the hospital as a random effect. Directed acyclic graphs were used to drive the selection of variables to fit the regression model.

**Results:**

Patients with severe PAD had longer QTc than those without. Based on the multivariable mixed-effect logistic regression model, a prolonged QTc was positively associated with severe PAD (odds ratio (OR) = 2.61; 95% CI: 1.07–6.35) and severe DFUs (Wagner grade score ≥ 3) (OR = 2.87; 95% CI: 1.42–5.81).

**Conclusions:**

A prolonged QTc was associated with severe PAD in patients with DFUs. Further research is required to ascertain whether the association is causal.

## Introduction

Diabetic foot disease, a common complication in patients with diabetes mellitus (DM) (1), is generally associated with a high prevalence of severe macroangiopathic comorbidities that result in high morbidity and mortality (2). About 49% of patients with DM and foot ulcers (DFUs) have coexisting peripheral artery disease (PAD) (3), which is usually asymptomatic. For these patients, the coexisting sensory neuropathy may mask the symptoms of intermittent claudication and ischemic rest pain. An earlier assessment of the severity of PAD is required to determine the need for revascularization to promote ulcer healing in patients with DFUs (4).

The QT interval, defined as the time between the beginning of the Q wave and the end of the T wave, represents the duration of electrical depolarization and repolarization of the ventricular walls (5). The prolongation of heart rate-corrected QT interval (QTc) is a possible consequence of cardiac autonomic neuropathy, and it is a predictor of lethal arrhythmias (6, 7). It has been demonstrated that patients with DM have a more frequent occurrence of QTc prolongation than those without (8). Patients with DFUs and prolonged QTc tend to have a higher prevalence of severe DFUs (Wagner grade 3–5) (9).

Few studies have addressed the association of QTc interval with arterial disease (10, 11, 12, 13). A clinical study found an association between the carotid intima-media thickness and QTc prolongation in patients with type 2 DM (11, 13). A prolonged QTc was found to be associated with PAD in the general population (10), but no study to date has identified a link between prolonged QTc and the severity of PAD in patients with DFUs.

It is important to understand the potential risk factors of the severity of PAD in patients with DFUs; this might be helpful for early identification and subsequent therapy initiation for patients with severe PAD. Therefore, this study was performed to assess the association of prolonged QTc with the risk of severe PAD in patients with type 2 DM and foot ulcers based on duplex ultrasonography.

## Materials and methods

### Study population

The study participants were patients diagnosed with DFUs according to the 2015 International Working Group on the Diabetic Foot diagnostic criteria (14) in one of the medical institutions in Wenzhou city, China (The First Affiliated Hospital of Wenzhou Medical University, Wenzhou People’s Hospital, Rui’an People’s Hospital, Rui’an Traditional Chinese Medicine Hospital, and Pingyang People’s Hospital). A total of 472 patients were consecutively enrolled from October 2015 to September 2019. The exclusion criteria included those with previous coronary heart disease, congenital long QT syndrome, pacemaker rhythms, atrial fibrillation, other types of arrhythmias, and hypokalemia before or during hospitalization, and persons under medication affecting the QTc interval (antiarrhythmic, beta-blocker, digitalis, quinolone antibiotics, and psychotropic drugs) within the past 6 months (15). Patients with an abnormal heart rate (<60 or >100 bpm) within 24 h of admission were excluded. A total of 281 patients with DFUs were included in this study.

The study protocol was approved by the ethics committee of the First Affiliated Hospital of Wenzhou Medical University. A written informed consent was obtained from all patients prior to their participation.

### Data collection and measurements

All patients were assessed by diabetologists and diabetic care nurses. The patient's history included information about demographic characteristics, DM duration, coronary heart disease, hypertension, and lifestyle habits, including smoking, and alcohol use. The resting blood pressure was also recorded. Hypertension was defined as the use of hypertensive medications or a mean resting systolic blood pressure (SBP) ≥ 140 mmHg or diastolic blood pressure (DBP) ≥ 90 mmHg. Smoking was categorized as currently smoking or non-smoking (including ex-smokers). Alcohol use was categorized as currently drinking or no alcohol consumption. Diabetes mellitus therapy was categorized as (i) oral hypoglycemic agent (OHA) therapy only, (ii) insulin therapy only, (iii) both OHA and insulin therapy, and (iv) none or unclear. The laboratory parameters, including high-density lipoprotein cholesterol (HDL-C), low-density lipoprotein cholesterol (LDL-C), total cholesterol, triglyceride (TG), hemoglobin A1c (HbA1c), albumin (ALB), hemoglobin (Hb), and creatinine, were measured using the standard protocols. The estimated glomerular filtration rate (eGFR) was calculated according to the Chronic Kidney Disease Epidemiology Collaboration (CKD-EPI) equation (16).

### Definition and grouping

Both lower extremity arteries (femoral artery, superficial femoral artery, popliteal artery, anterior tibial artery, posterior tibial artery, and dorsalis pedis artery) of patients were examined by duplex ultrasonography for intima-media thickness, plaque occurrence, stenosis, blood vessel diameter, and filling defects in blood flow. Vascular stenosis was defined as the presence of any stenosis ≥ 50% of the lumen of lower extremity arteries. Occlusion was defined as the total obliteration of the lumen (absence of blood flow) in any segment of the lower extremity arteries. PAD was defined by qualified, experienced ultrasonic diagnostic experts. The degree of severity of PAD based on the duplex ultrasonography was classified as no severe PAD group (without stenosis or occlusion) and severe PAD group (with stenosis or occlusion). The degree of severity of DFUs was subdivided according to the Wagner classification (17) (grade score 0–5) and categorized as no severe DFUs group (Wagner grade score < 3) and severe DFUs group (Wagner grade score ≥ 3).

### QTc measurement

All patients had a standard 12-lead resting ECG recorded within 24 h of admission. The ECGs were reviewed for rhythm analysis and measured by two independent cardiologists. The QT interval was calculated from the beginning of the QRS complex to the end of the T wave. Then, the value was corrected for heart rate according to the Bazett formula: QTc = QT interval/square root of the RR interval (QTc = QT/[RR]1/2). A QTc interval ≥ 450 ms in men or ≥460 ms in women was defined as prolonged QTc. A QTc interval < 450 ms in men or <460 ms in women was categorized as no prolonged QTc. To prevent the overestimation or underestimation of the QTc, patients with abnormal heart rates (<60 or >100 bpm) were excluded.

### Statistical analysis

The one-sample Kolmogorov–Smirnov test was used to examine the normality of the distributions. The data are presented as *n* (%), mean ± s.d., or in the case of skewed variables, as median and interquartile range. A multivariable-adjusted logistic regression analysis using a mixed-effect model was performed. A random effect by the hospital was introduced to account for the clustering of patients at the hospital level. Directed acyclic graphs (DAGs) (Supplementary Figs 1and 2, see section on supplementary materials given at the end of this article) were constructed using an online DAGitty software (http://www.dagitty.net) to assess the minimally sufficient sets of variables (18), which included age, sex, hypertension, smoking, alcohol use, DM duration, eGFR, LDL, and HbA1c for severe PAD. The variables for severe DFUs were age, sex, smoking, alcohol use, DM duration, diabetic foot duration, eGFR, LDL, and HbA1c. The adjusted odds ratio (OR) and 95% CI were calculated. The statistical analyses were performed using SPSS (IBM, IL, USA) version 22 and R (3.6.0, R Core Team).

## Results

### Clinical characteristics of the study population

Based on the duplex ultrasonography, 122 out of 281 patients with DFUs (43.4%) had severe PAD. The clinical characteristics of the study population are shown in Table 1. Patients with severe PAD had longer diabetic foot duration, higher prevalence of hypertension, longer QTc, and lower eGFR than those without.

**Table 1:** Baseline characteristics of participants with and without severe PAD.

Characteristic	No-severe PAD (*n* = 159)	Severe PAD (*n* = 122)
Male (%)	106 (66.7)	71 (58.2)
Age (years)	66 (56–74)	72 (66–80)
Smoking (%)	56 (35.4)	38 (31.1)
Alcohol use (%)	49 (30.8)	31 (25.4)
Diabetes duration (years)	10 (6–17)	10 (8–20)
Diabetic foot ulcer duration (days)	20 (7–30)	30 (10–60)
Severe DFUs (%)	66 (41.5)	58 (47.5)
Hypertension (%)	84 (52.8)	96 (78.7)
SBP (mmHg)	141 ± 22	146 ± 21
DBP (mmHg)	75 ± 12	76 ± 11
Creatinine (μmol/L)	69 (58–91)	77 (59–107)
eGFR (EPI) mL/min/1.73m^2^)	88.9 (67.4–100.3)	78.4 (50.3–94.5)
ALB (g/L)	34.4 (30.1–37.7)	34.9 (31.1–37.9)
Hb (g/L)	119.5 (104.8–133.3)	118.0 (105.5–128.0)
HbA1c (%)	9.5 (7.7–11.3)	9.0 (7.3–11.4)
TC (mmol/L)	4.2 (3.3–4.9)	4.11 (3.30–5.16)
TG (mmol/L)	1.22 (0.88–1.78)	1.33 (0.91–1.88)
HDL-C (mmol/L)	0.95 (0.75–1.19)	0.93 (0.75–1.10)
LDL-C (mmol/L)	2.29 (1.76–2.95)	2.37 (1.67–3.05)
DM therapy		
OHA therapy (%)	65 (40.9)	58 (47.5)
Insulin therapy (%)	46 (28.9)	35 (28.7)
OHA and insulin therapy (%)	34 (21.4)	21 (17.2)
None or unclear (%)	14 (8.8)	8 (6.6)
QTc interval (ms)	429 (407–445)	438 (423–455)

ALB, albumin; DBP, diastolic blood pressure; DM, diabetes mellitus; eGFR, estimated glomerular filtration rate; Hb, hemoglobin; HbA1c, hemoglobin A1c; HDL, high-density lipoprotein; LDL, low-density lipoprotein; OHA, oral hypoglycemic agent; PAD, peripheral arterial disease; QTc, heart rate-corrected QT interval; SBP, systolic blood pressure; TC, total cholesterol; TG, triglyceride.

### Association between prolonged QTc and severe PAD, severe DFUs

The unadjusted and multivariable-adjusted mixed-effects logistic regression analyses were performed to evaluate the relationship between prolonged QTc and severe PAD, severe DFUs (Table 2). The adjusted ORs for severe PAD and severe DFUs in prolonged QTc groups were 2.61 (95% CI: 1.07–6.35) and 2.87 (95% CI: 1.42–5.81), respectively.

**Table 2:** Association between prolonged QTc and severe PAD, severe DFUs in patients with DFUs.

	Unadjusted OR	95% CI	Adjusted OR	95% CI
Severe PAD				
No-prolonged QTc (Ref)	1.00		1.00	
Prolonged QTc	1.98	1.11–3.52	2.61^a^	1.07–6.35
Severe DFUs				
No-prolonged QTc (Ref)	1.00		1.00	
Prolonged QTc	2.48	1.38–4.45	2.87^b^	1.42–5.81

^a^The multivariable mixed-effects logistic regression was adjusted for risk factors including age, sex, hypertension, smoking, alcohol use, diabetes duration, eGFR, LDL, HbA1c as fixed effect and hospital as random effect. ^b^The multivariable mixed-effects logistic regression was adjusted for risk factors including age, sex, smoking, alcohol use, diabetes duration, diabetic foot duration, eGFR, LDL, HbA1c as fixed effect and hospital as random effect.

Ref, reference.

## Discussion

To our knowledge, this is the first study to reveal the associations between prolonged QTc and severe PAD in patients with DFUs based on duplex ultrasonography. In this study, a longer QTc was found in patients with DFUs and severe PAD. Prolonged QTc was significantly and positively associated with severe PAD based on multivariable logistic regression analyses in patients with DFUs.

Only few studies have focused on the QTc of patients with DFUs. Fagher *et al.* found that in patients with DFUs, QTc prolongation was associated with increased mortality (19, 20), and hyperbaric oxygen therapy might prevent QTc prolongation (21). Wang *et al.* (9) found that QTc prolongation was associated with a higher CVD mortality in patients with DFUs, but it cannot predict ulcer healing or recurrence. The present study extends the associations to the severity of PAD in patients with DFUs.

The mechanism underlying the association between prolonged QTc and severe PAD has not been fully elucidated. Atherosclerosis usually causes PAD. Other causes include inflammation (22), which can reduce the vascular nitric oxide bioavailability (23). It inhibits the Ca^2+^-ATPase and K^+^/Na^+^-ATPase, leading to an increase in cytosolic-free calcium and prolongation of myocardial repolarization (24). Accordingly, patients with severe PAD might have longer QTc. Patients with PAD have an equivalent cardiovascular risk compared to patients with previous myocardial infarction (25). Previous studies have indicated that QTc is associated with the risk of CVDs in different populations (26, 27, 28, 29). Patients with severe PAD are at a high risk for CVDs, which might affect the QTc. Studies have also indicated that QTc is associated with age (30), glycemic control (31), hypertension (8), hyperuricemia (32), hyperinsulinemia (33), and metabolic syndrome (34), which are the risk factors for the occurrence of PAD in patients with DM.

According to a previous study (9), which found that patients with DFUs and prolonged QTc tended to have a higher prevalence of severe DFUs (Wagner grade 3–5) in China, the multivariable regression analysis in this study found that a prolonged QTc was associated with severe DFUs; however, the underlying mechanism remains unknown. The severity of DFUs is affected by many factors, including other complications of DM and age. Which DM complication is most relevant to the severity of DFUs remains unclear (35). Studies have reported that QTc is associated with age (30), neuropathy, nephropathy, and multiplicity of microvascular complications (36). DM complications and age might play important roles in the relationship of prolonged QTc and severe DFUs.

This study has several strengths. First, PAD was diagnosed by more precise measurement, the duplex ultrasonography. Duplex ultrasonography is mostly used in detecting and localizing lesions in different territories of the vascular system and in quantifying the severity grade with the application of velocity and pressure gradient criteria. It can diagnose arterial diseases at a very early stage (37). Duplex ultrasonography is often recommended as the first-line non-invasive imaging for patients with PAD. It has 85–90% sensitivity and >95% specificity in detecting stenosis >50% (38). Secondly, the participants of this study were from different medical institutions. Therefore, the results may be applicable to general patients with DFUs. Thirdly, a random effect by the hospital was introduced to account for the clustering of patients at the hospital level. Fourthly, DAGs were used to guide the construction of multivariable logistic regression models. DAGs are transparent approaches to identify the confounding variables that rely on prior knowledge and assumed causal effects. DAGs can be preferred over the traditional methods (39).

In this study, the association between prolonged QTc and severe PAD was strengthened after adjusting for confounding variables suggested by DAGs. Confounding variables which may attenuate the association seem to be distinguished by DAGs. However, which variables play the critical roles remains unclear. The strengthened associations after adjustment have also been found in other studies. A previous study found that the association between diabetes and Alzheimer's disease was strengthened after adjusting for cardiovascular diseases (40). Sakhamuri *et al.* found that the association of a low forced vital capacity with a low BMI was strengthened in the adjusted model. In their view, this suggested a more direct association (41).

This study also has a limitation. The observed associations between QTc and severe PAD in patients with DFUs have limited causal inference given the nature of the study design.

## Conclusion

This study demonstrated an independent positive association between prolonged QTc and the severity of PAD in patients with DFUs. Further studies are required to ascertain whether a prolonged QTc may help identify patients with DFUs who are at risk of severe PAD.

## Supplementary Material

Supplemental Figure 1

Supplemental Figure 2

## Declaration of interest

The authors declare that there is no conflict of interest that could be perceived as prejudicing the impartiality of the research reported.

## Funding

This work was supported by grants from the Wenzhou Municipal Science and Technology Bureau http://dx.doi.org/10.13039/501100007194 (Y20180609).

## Ethics approval and consent to participate

The study protocol was approved by the ethics committee of the First Affiliated Hospital of Wenzhou Medical University. Written informed consent was obtained from all patients prior to their participation.

## Availability of data and materials

The datasets used and analyzed during the current study are available from the corresponding author on reasonable request.

## Author contribution statement

J H: software, investigation, writing-original draft. W-Y L: software, investigation. X H: writing – review and editing. F-F J: data curation. Z-R X: data curation. F L: data curation. F-X S: supervision. H Z: conceptualization, writing – review and editing. All authors read and approved the final manuscript.
