# Supplementary figures and images for: Association between heart rate-corrected QT interval and severe peripheral arterial disease in patients with type 2 diabetes and foot ulcers
Source: Endocr Connect. 2021 Jul 5;10(8):845–51. doi: 10.1530/EC-21-0140 (PMC8346192; doi:10.1530/EC-21-0140)

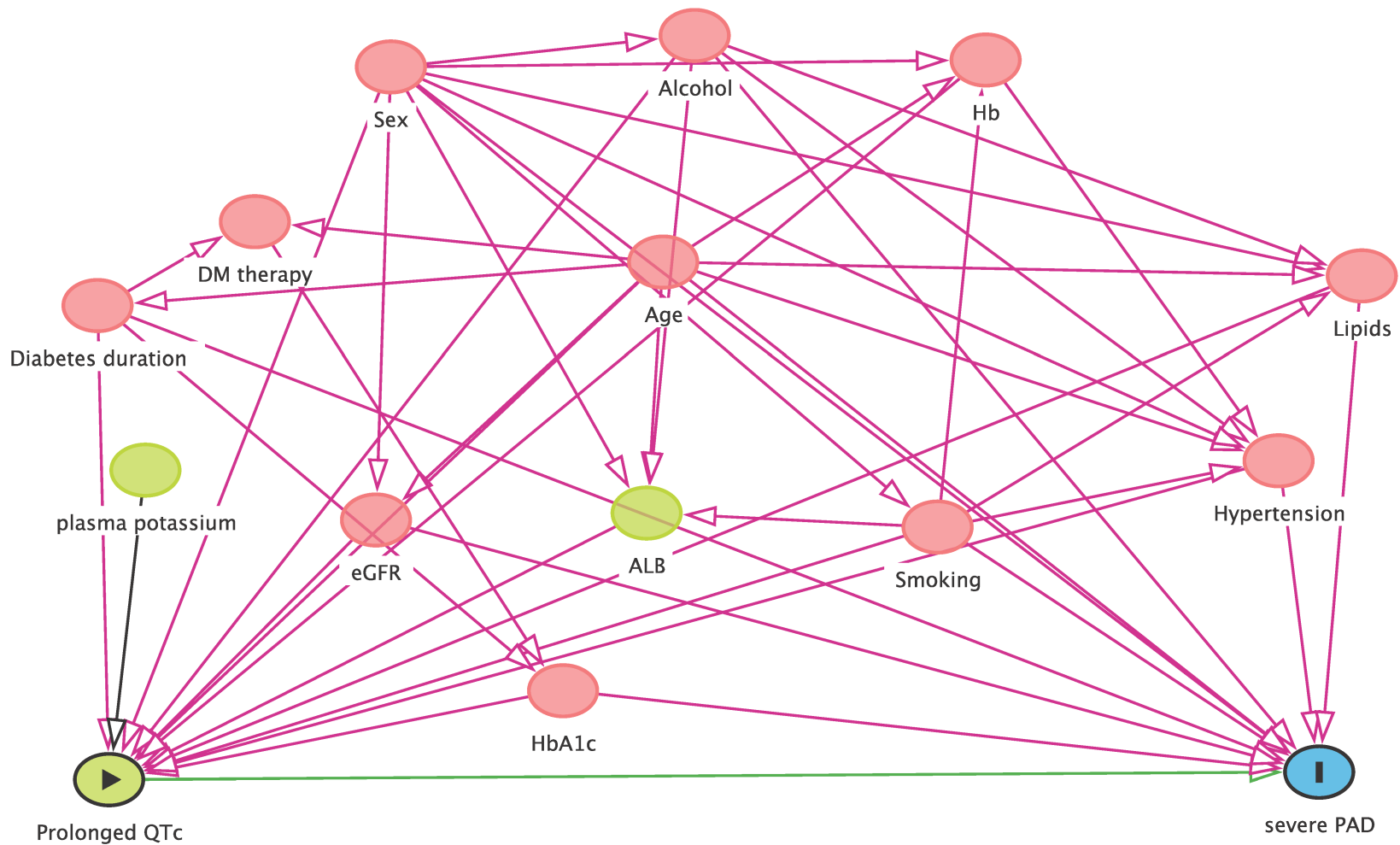

Supplement: Supplemental Figure 1 [file supplementary_figure_1.pdf]

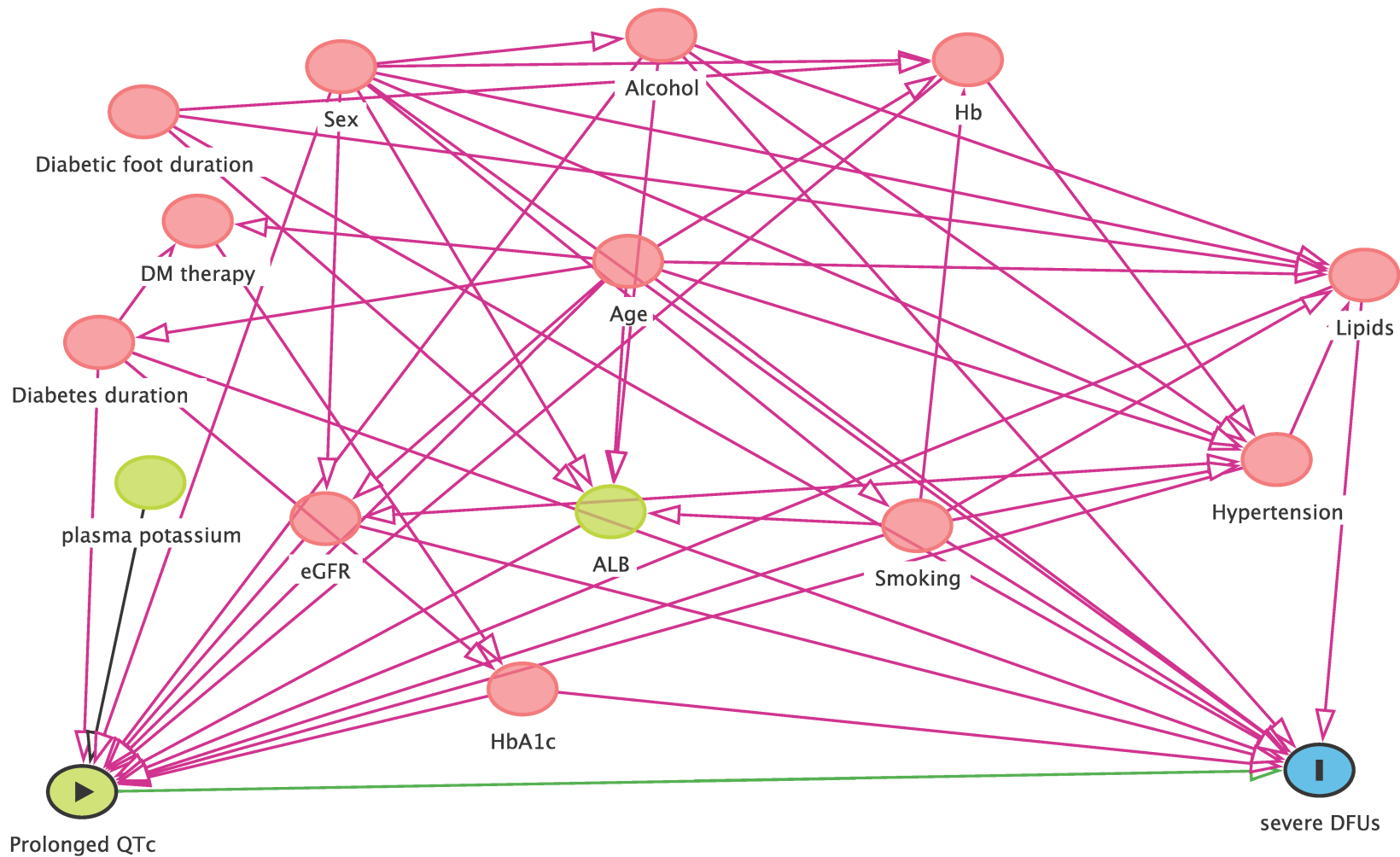

Supplement: Supplemental Figure 2 [file supplementary_figure_2.pdf]
